# What Is Solastalgia and How Is It Measured? SOS, a Validated Scale in Population Exposed to Drought and Forest Fires

**DOI:** 10.3390/ijerph192013682

**Published:** 2022-10-21

**Authors:** Cristian Cáceres, Marcelo Leiva-Bianchi, Carlos Serrano, Yony Ormazábal, Carlos Mena, Juan Carlos Cantillana

**Affiliations:** 1 Laboratory of Methodology, Behavioral Sciences, and Neurosciences, Faculty of Psychology, Universidad de Talca, Talca 3460000, Chile; 2 Faculty of Social Sciences and Humanities, Autonomous University of Chile, Talca Campus, Talca 3460000, Chile; 3 Faculty of Economics and Business, Universidad de Talca, Talca 3460000, Chile; 4 Faculty of Administration and Economics, Universidad Tecnológica Metropolitana, Santiago 8320000, Chile

**Keywords:** PTSD, SOS, climate change, *solace*, *algia*, environment

## Abstract

Solastalgia is a recent concept that refers to disruptive psychological responses in people exposed to environmental degradation. The aim of this study was to determine the number of dimensions solastalgia has using a sample of people exposed to the effects of climate change in the coastal dry land of Maule region, Chile. In order to achieve this, a Scale Of Solastalgia (SOS) was designed and then validated, by means of applying it to 223 inhabitants at the municipalities of Pencahue (*n* = 105) and Curepto (*n* = 118), who were also evaluated by the Short Post-traumatic Stress Disorder Rating Interview (SPRINT-E). Using robust validation methods (Parallel factor analysis and Omega), two dimensions were obtained for solastalgia: *solace* and *algia*. Both correlate with the SPRINT-E scale (r = 0.150, *p* < 0.01 and r = 0.359, *p* < 0.01, respectively) and have 58% sensitivity and 67% specificity to detect cases of post-traumatic stress disorder (PTSD). Like PTSD, solastalgia is related to psychopathologies expected after disasters and also presents a spatial pattern where the concentration of positive cases occurs in places of greater exposure to environmental change or degradation.

## 1. Introduction

The concept of solastalgia was conceived by Albrecht [1,2] to refer to the disruptive psychological responses presented by people exposed to environmental degradation. Negative changes in environmental conditions disconnect people from their surroundings, which can lead to distress [3], as well as a lack of comfort, identity, and attachment to home [4]. In this way, solastalgia can be described as a deep nostalgia suffered by people who live in territories, communities, or homes that have become degraded and different to what they were accustomed to [5]. It is composed of the Latin concept of *solace* (comfort or consolation provided by the physical home [6]) and the Greek *algia* (pain [5]). This definition includes two dimensions of the concept: (1) desolate because of the degradation of the environment where an individual lives and (2) distress linked to this degradation [5,7]. Therefore, the concept of solastalgia can be understood as the distress caused by a change in an appreciated place and its cumulative impact on the mental health of those who live in that specific location. Psychologically, since people feel attached to the place they inhabit and consider it valuable, the process of watching it degrade will cause them distress. The previous could be related to the ecological pain caused by the loss of species, ecosystems, and landscapes. A close concept is eco-anxiety, which is understood as the anxiety produced by living in a changing and uncertain environment [8].

Although the term solastalgia has been frequently mentioned in recent years, its operational definition is not entirely clear, and different variations can be found. The first measurement of solastalgia was taken by Higginbotham et al. [3] who studied the population of a territory widely affected by mining activities (Towns of Singleton and Dingog, Upper Hunter, Australia), obtaining a single dimension through principal component analysis (PCA). Subsequently, the validity of the construct was evaluated in a study of a population exposed to an eruption of the Merapi volcano in Indonesia [9]. From this study, three dimensions were obtained, and two of them were very similar to those defined theoretically: *melancholy* and *solace*. In this study, as in the one by Higginbotham et al., PCA was used; however, its application is not recommended because results may have fewer dimensions than the ones obtained from more robust methods [10,11,12]. Nevertheless, its third dimension (lack of control) contains only one item, which is insufficient to define a robust dimension [11] and reinforces the idea of the two-dimensionality of the construct. In another case, the Environmental Distress Scale (EDS) was applied to a population exposed to a forest fire in Wallow, AZ, USA [13], where PCA was again used to conclude that solastalgia has only one dimension.

Recently, two studies report that solastalgia is a one-dimensional construct. In the first [14], a scale was applied to a population exposed to the loss of beach territory caused by coastal erosion in the southeast of Ireland, assessing the reliability of two dimensions that were not normally distributed. When this occurs, a robust procedure is to apply a parallel exploratory factor analysis using polychoric correlations [15]. However, none of these procedures were performed in the study. In the second case [16], two scales (one with 8 and the other with 13 items) were applied to a population exposed to the construction of reservoirs for electricity generation. In this case, PCA was also used, which supports the conclusion of solastalgia having only one dimension.

Considering these efforts, it is necessary to elucidate the number of dimensions that define solastalgia. Based on the review published by Galway et al. [7], the present article aims to clarify this question. To achieve this goal, a Scale Of Solastalgia (SOS) was designed and then validated through its application to the population in the dry inner zone of the Maule region, Chile, which has been exposed to various environmental changes throughout the last 20 years [17]. Economic reasons have led to the introduction of massive pine and eucalyptus plantations in this territory, drastically changing the landscape and the conditions thereof. This situation has been aggravated by the effects of climate change, causing a drought that has lasted more than 10 years in the area, thus generating conditions for the occurrence of the largest forest fires recorded in Chile in recent times [18].

## 2. Materials and Methods

### 2.1. Elaboration of the Scale

Scientific articles from the Proquest, Scopus, Ebsco, and WoS databases that include the word solastalgia in their title were identified. In each case, scales were used, and the nine most frequent items were identified. The wording of these items was adapted for general contexts and then submitted to the review and evaluation of five experts in climate change, psychosocial impact, psychometrics, forestry sciences, and geomatics. As a result of the evaluation, two of the items were eliminated and three new ones were added to incorporate the *algia* dimension (proposed in the original definition by Connor et al. [4]), linked to the pain and distress generated by environmental changes in people. Thus, a preliminary version of a Scale Of Solastalgia (SOS) was obtained, composed of 11 items evaluable on an ordinal scale from 0 (strongly disagree) to 3 (strongly agree).

### 2.2. Application of the Scale

**Figure 1:**
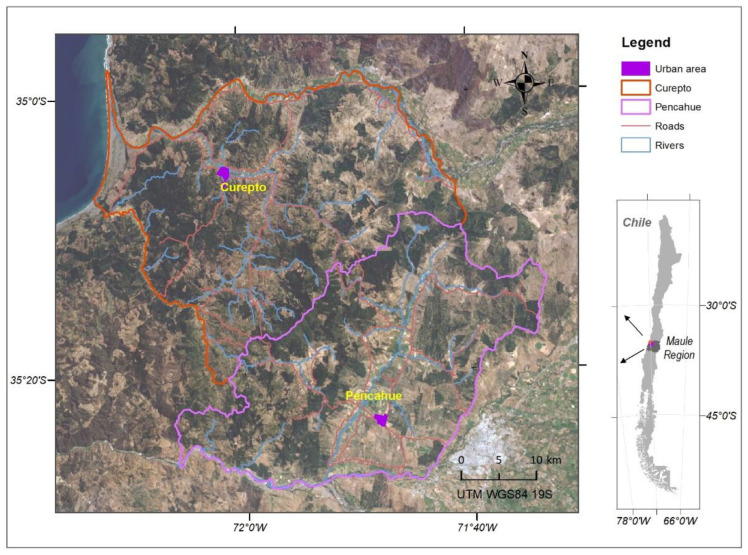
Localization of the study area in Chile.

The SOS was applied to 223 people over 18 years of age (58% women), inhabitants of the municipalities of Pencahue (*n* = 105) and Curepto (*n* = 118). These locations (Figure 1) have been exposed to large forest fires and prolonged droughts over the last 10 years. The selection of people was carried out through cluster sampling, considering two stages. First, the urban and rural inhabitants of each municipality were identified, according to the records provided by the 2017 Census of the National Institute of Statistics [19]. Then, sectors were selected according to the distribution of the population in the census districts of rural areas and in the blocks of urban areas, singling out 30% of the dwellings in each sector. The SOS was applied by trained interviewers, who read an initial statement, emphasizing the environmental context in which the respondent lives. The SOS was applied jointly with the SPRINT-E (short post-traumatic stress disorder rating interview) scale [20] to be used as the gold standard when determining the validity of the SOS. Finally, the geographical location of each evaluated individual was recorded using Garmin GPS receivers, model GPSMAP 65. This generated a point to which all the data obtained by the applied scales was subsequently linked.

### 2.3. Evaluation of the Scale

The data obtained with the SOS were evaluated by means of parallel factorial analysis (PARAFAC). This method is recommended in such cases due to the robustness it offers regarding the identification of dimensions (factors) in ordinal data [21]. Having verified the non-compliance with the assumption of expected multivariate normality (Sk and Ku; *p* < 0.05) for ordinal data from social and behavioral sciences [22], the use of a polychoric correlation matrix [11] was decided upon. To determine the adequacy of the correlation matrix, the Bartlett sphericity test (*p* < 0.05) was used jointly with the Kaiser-Meyer-Olkin coefficient (KMO ≥ 0.8). To determine the number of dimensions, regardless of the distribution, the classical implementation [23], the extraction method of unweighted least squares (ULS), the oblimin rotation, and the simulated values with the 95th percentile [15,24] were used. With this, the proper number of dimensions is found when the difference between the simulated and observed eigenvalues is minimal and positive. Thus, from the matrix of rotated factors, the items that met at least one of the following conditions were eliminated: weights (w) less than 0.40 in the factor where they contributed the most; cross loads more than 0.30 in the other factors; and differences less than 0.20 with the other items [25]. Gamma-GFI (GFI > 0.8 [26]) and root mean square residual (RMSR ≤ 0.0673 [24]) were used as adjustment indicators. In addition, the percentage of accumulated variance was reported [27]. The reliability of the SOS and its dimensions were obtained by the coefficients Cronbach’s Alpha (α > 0.7) and McDonald’s Omega (ω > 0.7). The latter statistic is more stable, reflects the true level of reliability, and does not depend on the number of items [28,29].

### 2.4. Validation of the SOS

The validity of the scale was evaluated using Pearson’s correlations (r; *p* < 0.01) between the totals and dimensions of the SOS and SPRINT-E. It should be noted that SPRINT-E is an instrument that has demonstrated validity, reliability, and accuracy to evaluate two dimensions: Post-traumatic Stress Disorder (PTSD) symptoms and reactions produced by disasters (for example, earthquakes, droughts, and forest fires, among others). SPRINT-E considers 12 items to evaluate the re-experimentation of the event, hyper-activation, avoidance/numbing, adaptation to daily life, alcohol/drug use, and suicidal ideation. Each item is measured with values from 0 (minimum level) to 3 (maximum level), giving a maximum total of 36 points. When an item has a score of 2 or 3, it is considered an intense symptom. When a person has three or more severe symptoms, it is considered a case of PTSD.

The accuracy of the SOS was evaluated considering the degree of coincidence between the total SOS score and the PTSD cases (obtained with SPRINT-E), using ROC (Receiver Operating Characteristic) curves. The SOS is expected to be sensitive (sensitivity > 0.6) and specific (false-positive < 0.1) to find PTSD cases. For the analysis, the bootstrapping method (with 1000 repetitions) was applied in the MedCalc software (version 20.010, MedCalc software Ltd., Ostend, Belgium). Here, the larger the area under the curve (AUC; *p* < 0.05), the greater the accuracy of the SOS, having levels of: excellent (0.9 to 1), good (0.7 to 0.8), regular (0.6 to 0.7), poor (0.5 to 0.6), and deficient (less than 0.5 points). The analysis also made it possible to identify the optimal cut-off points using the Youden index for the total SOS and its dimensions [30,31]. For analyzing the cases of PTSD and SOS, according to their territorial location and physical environment, thematic maps were elaborated. These maps were based on the location registered for each person evaluated and the results obtained for the evaluations plus context elements, such as communal boundaries, roads, rivers, and a satellite image background.

## 3. Results

**Figure 2:**
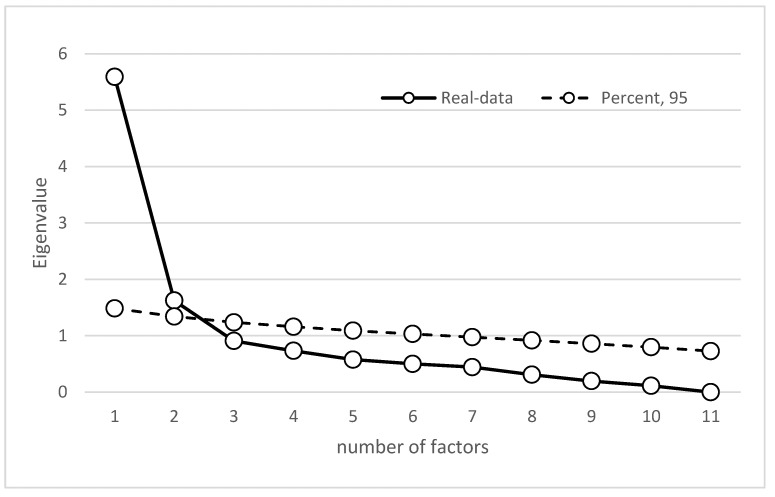
Scree plot with eigenvalues for real, average, and simulated data.

The polychoric correlation matrix was adequate (KMO = 0.890; Bartlett’s = 2492.5; *p* < 0.01) as the basis for parallel factor analysis by verifying non-compliance with the assumption of multivariate normality (Sk = 152.138; *p* > 0.05; Ku = 392.139; *p* < 0.05). The result of the parallel analysis using the 95th percentile method indicates that the SOS would have two dimensions since, in the second factor, the difference was minimal and positive between the simulated and observed eigenvalues (Table 1 and Figure 2). This solution explains 66% of the common variance and has excellent model fit indicators (GFI = 0.988; RMSR = 0.0625).

The analysis of the rotated factors matrix (Table 2) indicates that all items have loads greater than 0.3 in their respective factor. However, item 8 “I fear a catastrophe is approaching” has a difference of less than 0.2 between factors, so it was eliminated from the SOS. In this way, the SOS was composed of 10 items and two dimensions. Excellent reliability indicators were obtained for both the total SOS (α = 0.901; ω = 0.903) and the dimensions *solace* (α = 0.87; ω = 0.88) and *algia* (α = 0.908; ω = 0.908).

Table 3 shows that the total scores of the SOS and SPRINT-E scales have an adequate and significant correlation (r = 0.308; *p* < 0.01). The same occurs between the total SPRINT-E and the *solace* and *algia* dimensions, in which the correlation with the last one (r = 0.359; *p* < 0.01) is higher. The dimensions of PTSD symptoms (r = 0.330; *p* < 0.01) and reactions to disasters (r = 0.332; *p* < 0.01) also have a significant correlation with the *algia* dimension of the SOS.

**Figure 3:**
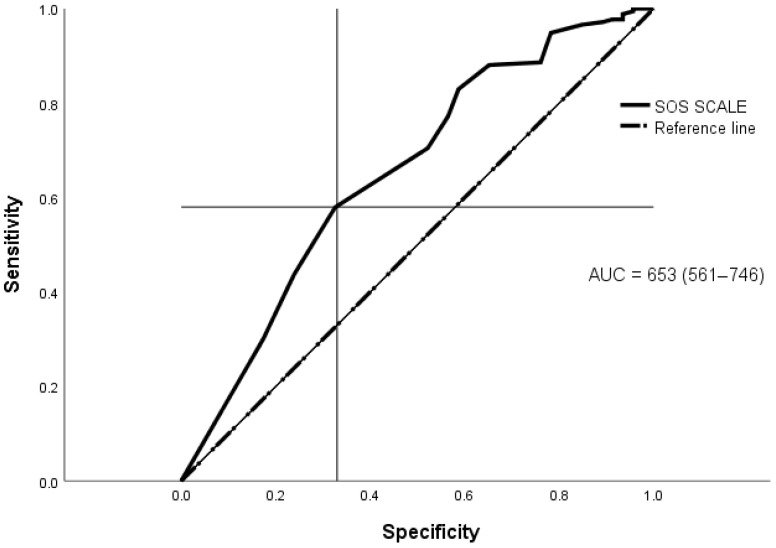
Curve ROC for total SOS.

These appropriate correlations corroborate the use of the SPRINT-E as the accuracy standard for the SOS. For a number of 176 (79.28%) observed cases of PTSD, SOS shows 58% of sensitivity and 67% of specificity in its detection. Therefore, its accuracy is regular (AUC = 0.653; *p* < 0.01; CI = 0.568–0.739), with a Youden index [J] = 0.2535 (CI 95% = 0.09 to 0.36) and an optimal cut-off score of 27 points (CI = 24–29) (Figure 3).

**Figure 4:**
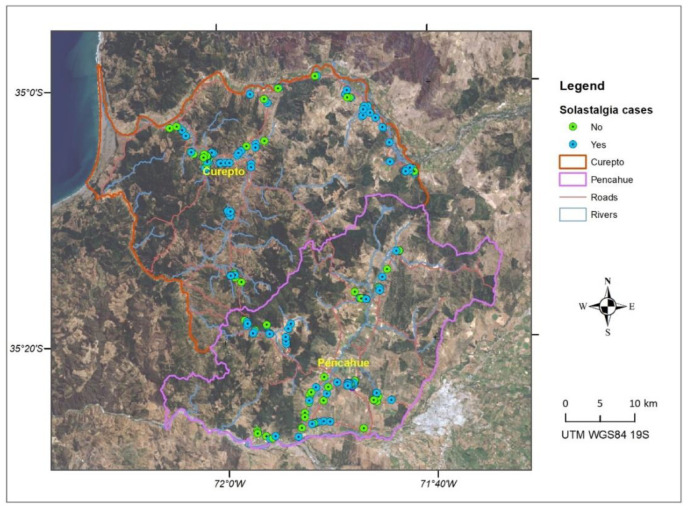
Spatial distribution of solastalgia cases.

**Figure 5:**
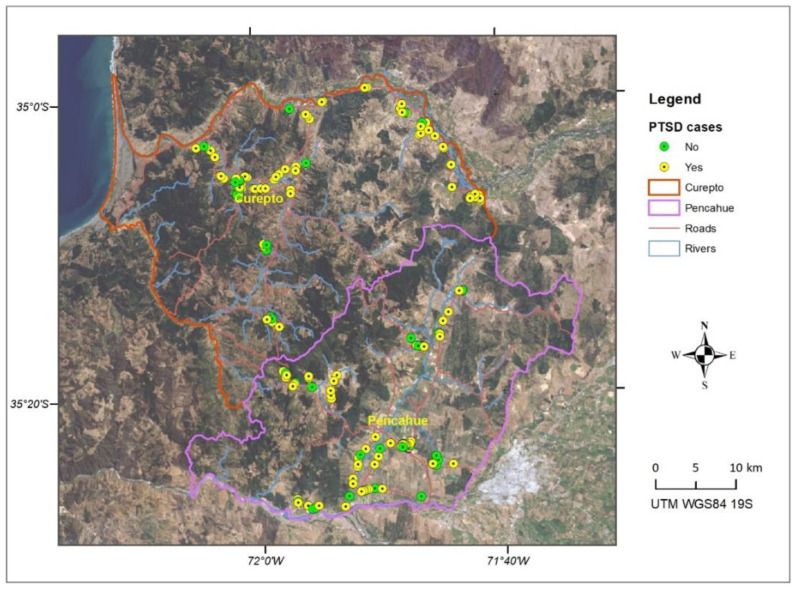
Spatial distribution of PTSD cases.

Figure 4 and Figure 5 show the spatial distribution of cases detected for solastalgia and PTSD. It is possible to appreciate a greater concentration of positive cases in communal areas more exposed to drought phenomena, such as the banks of the Mataquito River in the Northeast of Curepto and in agricultural valleys, where a decrease in flows and groundwaters have impacted agricultural and livestock activities. In turn, a concentration of positive cases in mountain areas can be seen, where multiple forest fires have affected forestry activity, such as the south of Curepto and the west of Pencahue.

**Figure 6:**
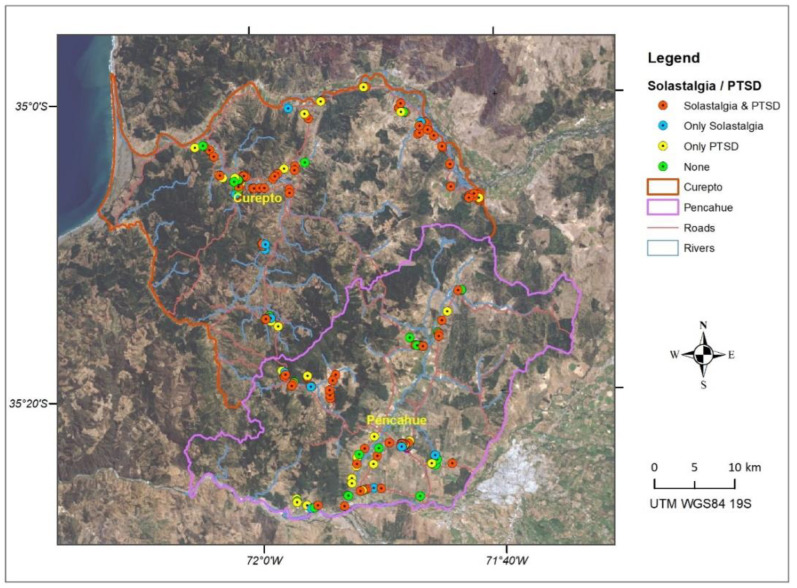
Spatial distribution of integrated solastalgia and PSDT cases.

**Figure 7:**
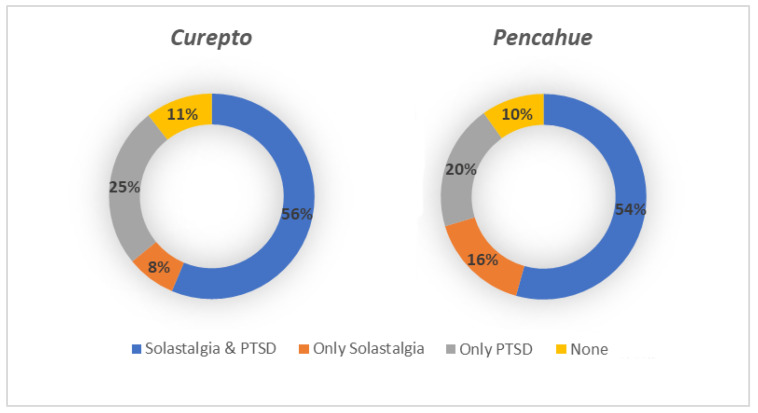
Percentage distribution of solastalgia and PSDT cases by location.

When observing both maps, a similar pattern in the spatial distribution of solastalgia and PTSD cases can be noted, which is expected, given the high level of correlation between both scales. Figure 6 shows the integration of the solastalgia and PTSD cases, where the spatial pattern of cases detected is more concentrated in Curepto than in Pencahue, and which would be related to the lack of water availability, in an area that greatly depends on this resource. It is worth mentioning that, on the one hand, Curepto is located between the coastal plain and the coastal mountain range, and it is an area that has suffered more significantly from the drought phenomenon. On the other hand, the municipality of Pencahue is located further inland, and benefits from the greater implementation of irrigation systems and channels, so its inhabitants have better endured the situation. Figure 7 shows that, in terms of proportion, the most important difference between the locations lies in the cases presenting only solastalgia or PTSD. It is evident that Curepto has a higher percentage of only PTSD cases than Pencahue because its inhabitants have faced more extreme conditions in terms of drought and forest fires, both situations that are more closely related to PTSD.

## 4. Discussion

The operational definition of solastalgia, validated by the SOS, coincides with Albrecht’s original proposal [1,2]. It has the dimensions of *solace* (comfort or consolation provided by the physical home) and *algia* (pain). This finding is new in the existing scientific literature since most studies evaluate solastalgia with a single dimension [13,14,16]. Only the study by Warsini et al. [9] accounts for three dimensions: *melancholy*, *solace*, and *loss of control*. It should be noted that the latter is composed of a single item, which is insufficient to define a dimension [11]. A robust analysis like the one applied in the present study could elucidate whether the loss of control item can be included within the melancholy dimension (equivalent to *algia*). It is likely that this will not happen because the *algia* dimension is linked more to emotions (pain, distress, anxiety) [5,7] and not to behaviors.

This emotional connection directly relates solastalgia with ecological pain and the eco-anxiety of living in a changing and uncertain environment [8]. For this reason, the SOS is presented as a useful tool to assess the mental health of people exposed to degraded environments. This is evidenced by the fact that both the total and the dimensions of the SOS were related to the SPRINT-E scale, which has proven to be a valid and accurate instrument for assessing PTSD after disasters [20]. The relationship between SOS and PTSD is consistent with what is found in communities exposed to environmental changes (for example, mining operations, climate change effects, and droughts), where people respond with feelings of fear, anxiety, uncertainty, isolation, stress, distress, or mental health problems in general [32,33,34]. In this regard, recent models of the psychosocial impact of disasters could explain why more disruptive responses (for example, Solastalgia, PTSD) are expected in exposure contexts [35]. It is worth mentioning that the SOS does not replace the use of more precise instruments to assess PTSD or other symptoms expected after disasters. However, scores equal to or greater than 27 on the SOS are an alert that should not be ignored. For future research, it is suggested that the items of the *algia* dimension refer explicitly to the environmental changes of the home, place, or landscape in which the person lives. That is, it is suggested to apply items 9, 10, and 11 of Table 2, adding the phrase “… due to changes in my environment”. Although the original wording was not a problem for the validity of the SOS, this suggestion points to a greater understanding of the items by those who answer them. This would continue to point to the concept of “desolation” (caused by the loss of the familiar landscape) originally proposed by Albrecht [1] and which for SOS corresponds to the *solace* dimension.

## 5. Conclusions

Solastalgia can be defined as the nostalgia, anxiety, stress, and worry of people living in degraded environments. The *solace* dimension refers to the loss of comfort or consolation provided by an inhabited environment and the nostalgia that its degradation causes to the exposed person. For its part, *algia* is understood as the set of emotions typical of living in degraded environments (for example, anxiety, stress, and worry). Like PTSD, solastalgia is related to the psychopathologies expected after disasters, so it is a useful indicator to alert authorities about risk situations for inhabitants exposed to degraded environments. In addition, positive cases of the solastalgia spatial distribution pattern concentrate on places with greater exposure to environmental changes or degradation. This should be studied further, in order to improve the detection and management of positive cases of solastalgia in populations directly exposed to altered, degraded, or lost environments. Finally, it is suggested to evaluate the validity and accuracy of the SOS in populations exposed to other environmental degradation events. Additionally, this study recommends using other robust analysis techniques such as Exploratory Structural Equation Modeling (ESEM), Latent Class Analysis (LCA), and factorial invariance analysis.

## Figures and Tables

**Table 1:** Factors for real and simulated data.

Factor	Real-Data Eigenvalue (RDE)	Percentile of Random Eigenvalue 95 (PRE95)	Difference between RDE and PRE95
1	5.59002	1.48486	4.10516
2	1.62464	1.34195	0.28269
3	0.90805	1.23716	−0.32911
4	0.73347	1.16031	−0.42684
5	0.57685	1.08981	−0.51296
6	0.50205	1.03270	−0.53065
7	0.44373	0.97459	−0.53086
8	0.31014	0.91877	−0.60863
9	0.19683	0.85967	−0.66284
10	0.11415	0.79793	−0.68378
11	0.00009	0.72816	−0.72807

**Table 2:** Rotated factor matrix.

	Item	*Solace*	*Algia*
1.	I feel sad when I see the landscape degraded or deteriorated.	0.701	0.115
2.	My lifestyle is threatened by water scarcity brought about by climate change.	0.542	0.172
3.	I feel nostalgic about losing the valuable elements of the place I live in (for example, clean air, water, landscape, peace and quiet).	0.999	−0.045
4.	It makes me sad that animals and plants are disappearing.	0.762	−0.028
5.	It makes me sad to think that one day I will be forced to leave this place as a result of climate change.	0.715	−0.066
6.	It distresses me to see the effects of climate change (for example, fires, droughts) on the place I live.	0.515	0.219
7.	The place I live in has lost its inherent characteristics.	0.582	−0.066
8.	I fear a catastrophe is approaching.	0.450	0.409
9.	Lately, I feel anxious.	0.076	0.795
10.	Lately, I have been tense.	−0.086	1.028
11.	Lately, I have been worried.	0.146	0.738

**Table 3:** Pearson correlations for total and dimensions of SOS and SPRINT-E.

		1	2	3	4	5
1	Total SOS	1				
2	*Solace*	** 0.827				
3	*Algia*	** 0.828	** 0.370			
4	Total SPRINT-E	** 0.308	* 0.150	** 0.359		
5	PTSD Symptoms	** 0.290	* 0.150	** 0.330	** 0.844	
6	Reactions to disasters	** 0.281	* 0.133	** 0.332	** 0.963	** 0.668

*: *p* < 0.05; **: *p* < 0.01.

## Data Availability

The data presented in this study are available on request from the corresponding author. The data are not publicly available due to privacy.
